# Genetic evidence for algal auxin production in *Chlamydomonas* and its role in algal-bacterial mutualism

**DOI:** 10.1016/j.isci.2023.108762

**Published:** 2023-12-16

**Authors:** Victoria Calatrava, Erik F.Y. Hom, Qijie Guan, Angel Llamas, Emilio Fernández, Aurora Galván

**Affiliations:** 1 Departamento de Bioquímica y Biología Molecular. Campus de Rabanales y Campus Internacional de Excelencia Agroalimentario (CeiA3), Edificio Severo Ochoa, Universidad de Córdoba, 14071 Córdoba, Spain; 2 Department of Biology and Center for Biodiversity and Conservation Research, University of Mississippi, University, MS 38677-1848, USA

**Keywords:** Plant biochemistry, Microbiology, Plant biology

## Abstract

Interactions between algae and bacteria are ubiquitous and play fundamental roles in nutrient cycling and biomass production. Recent studies have shown that the plant auxin indole acetic acid (IAA) can mediate chemical crosstalk between algae and bacteria, resembling its role in plant-bacterial associations. Here, we report a mechanism for algal extracellular IAA production from L-tryptophan mediated by the enzyme L-amino acid oxidase (LAO1) in the model *Chlamydomonas reinhardtii*. High levels of IAA inhibit algal cell multiplication and chlorophyll degradation, and these inhibitory effects can be relieved by the presence of the plant-growth-promoting bacterium (PGPB) *Methylobacterium aquaticum*, whose growth is mutualistically enhanced by the presence of the alga. These findings reveal a complex interplay of microbial auxin production and degradation by algal-bacterial consortia and draws attention to potential ecophysiological roles of terrestrial microalgae and PGPB in association with land plants.

## Highlights

• *Chlamydomonas* produces auxin using an extracellular L-amino acid oxidase (LAO1)
• Auxin inhibits algal multiplication and chlorophyll degradation under N limitation
• *Methylobacterium aquaticum* can degrade auxin in the presence of *Chlamydomonas*
• Auxin mediates a mutualism between *Chlamydomonas* and *Methylobacterium aquaticum*

## Graphical abstract

**Figure:**
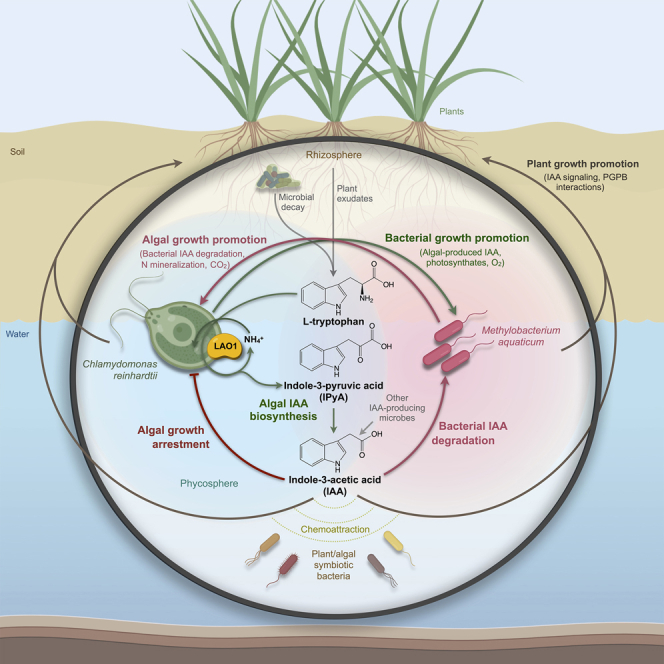

## Introduction

The auxin indole-3-acetic acid (IAA) is an essential signaling molecule that controls almost every aspect of plant development.^1^^,^^2^ Although initially thought to be produced exclusively by land plants, several studies have found that algae, bacteria, and fungi can not only respond to IAA but also produce it extracellularly, making it a widespread interkingdom signaling molecule.^1^^,^^3^^,^^4^^,^^5^^,^^6^^,^^7^^,^^8^

As in land plants, IAA plays a key role in controlling growth, photosynthetic activity, and primary metabolism of microalgae.^5^^,^^7^^,^^9^^,^^10^ Some bacteria produce IAA extracellularly to stimulate algal photosynthetic activity, primary metabolism, and growth.^5^^,^^9^^,^^11^^,^^12^ IAA can also be produced by green, red, and brown algae.^13^^,^^14^^,^^15^^,^^16^^,^^17^^,^^18^^,^^19^ Nevertheless, the molecular basis for *how* IAA is made in algae and its potential role in interkingdom interactions remains essentially unknown.^17^^,^^20^^,^^21^

The “phycosphere,” the algal analog of the plant rhizosphere, is the region immediately surrounding an algal cell that is enriched in exuded metabolites similar to those released by plant roots.^5^^,^^22^^,^^23^ These compounds include IAA and nutrients such as sugars and amino acids that serve ecological roles, are exchanged with bacteria, and act as signaling molecules essential for communication between taxa.^12^ There is mounting evidence of conspicuous similarities between the bacterial associations formed with algae and those formed with land plants^24^ (reviewed by Seymour et al.^25^). For instance, many bacteria that associate with algae, such as *Rhizobium* and *Sphingomonas*, are phylogenetically similar to those that form symbioses with land plants, suggesting broad eco-evolutionary affinities between bacteria and the diverse lineages of the plant kingdom.^24^^,^^26^^,^^27^ Some of these beneficial interactions have been exploited for biotechnological applications,^28^^,^^29^^,^^30^ notably toward improving sustainable agriculture.^31^^,^^32^ However, we still understand surprisingly little about both the ecology and the molecular mechanisms underlying most algal-bacterial interactions in nature.^25^^,^^33^^,^^34^

In prior work, we found that the model alga *Chlamydomonas reinhardtii*^35^ and plant-growth-promoting bacteria (PGPB) in the genus *Methylobacterium*^36^ can form mutualisms based on a carbon (C) and nitrogen (N) nutrient exchange.^37^ This beneficial interaction between *Chlamydomonas* and *Methylobacterium* can be harnessed to improve wastewater bioremediation, biomass generation, and hydrogen production.^38^ Briefly, these bacteria can mineralize exogenous amino acids and peptides that are poor N sources for *Chlamydomonas* to support the growth of the alga, which in turn provisions photosynthetic C sources like glycerol to promote bacterial growth.^37^ Notwithstanding, *Chlamydomonas* can grow axenically on most free amino acids and some short peptides using LAO1, an extracellular (periplasmic) L-amino acid oxidase (LAAO).^39^^,^^40^^,^^41^ *LAO1* is highly expressed in *Chlamydomonas* during N starvation and oxidizes L-amino acids present in the extracellular medium to produce ammonium, hydrogen peroxide, and corresponding keto acids^41^ (Table S1). Although ammonium is used to support algal growth, the resulting keto acids are surprisingly not used as a C source by the alga.^39^^,^^40^^,^^42^ We reasoned that although not metabolized by *Chlamydomonas*, these extracellular keto acids may provide some benefit and/or play some ecological role. For instance, some amino-acid-derived keto acids like α-ketoisocaproic (from L-leucine) or α-ketoisovaleric (from L-valine) show siderophore activity that may improve iron nutrition;^43^ others, like pyruvate (from L-alanine) and oxaloacetate (from L-aspartate), can scavenge hydrogen peroxide^44^ to potentially reduce oxidative stress; still others may serve as carbon sources for nearby organisms. Some LAO1-derived keto acids like indole pyruvic acid (IPyA, from L-tryptophan [L-Trp]) and phenyl pyruvic acid (PPA, from L-phenylalanine) are well-known precursors for auxin biosynthesis.^45^^,^^46^ Nonetheless, LAAO-mediated production of auxin has only recently been identified in a single bacterial species.^47^ We surmise that LAAO enzymes may play significant ecological roles in mediating cross-taxa interactions, particularly between microbes and diverse plant lineages, despite their relevance being largely neglected to date.

In this work, we present the first ever genetic evidence for auxin production in an alga, specifically the model alga *Chlamydomonas*. IAA is accumulated extracellularly under N limitation in an LAO1-dependent manner, expanding the role of LAAOs in microbial auxin production. We show the impact of extracellular IAA accumulation under N limitation on *Chlamydomonas* growth and its potential role in establishing beneficial interactions with methylobacteria. Our study reveals a new mode of algal-bacterial interaction mediated by algal-produced IAA that may have fundamental implications about the ecological impact of algae and PGPB in aquatic systems and terrestrial ecosystems potentially modulating plant responses.^48^

## Results

### *Chlamydomonas* produces IAA from tryptophan using the extracellular L-amino acid oxidase LAO1

**Figure 1:**
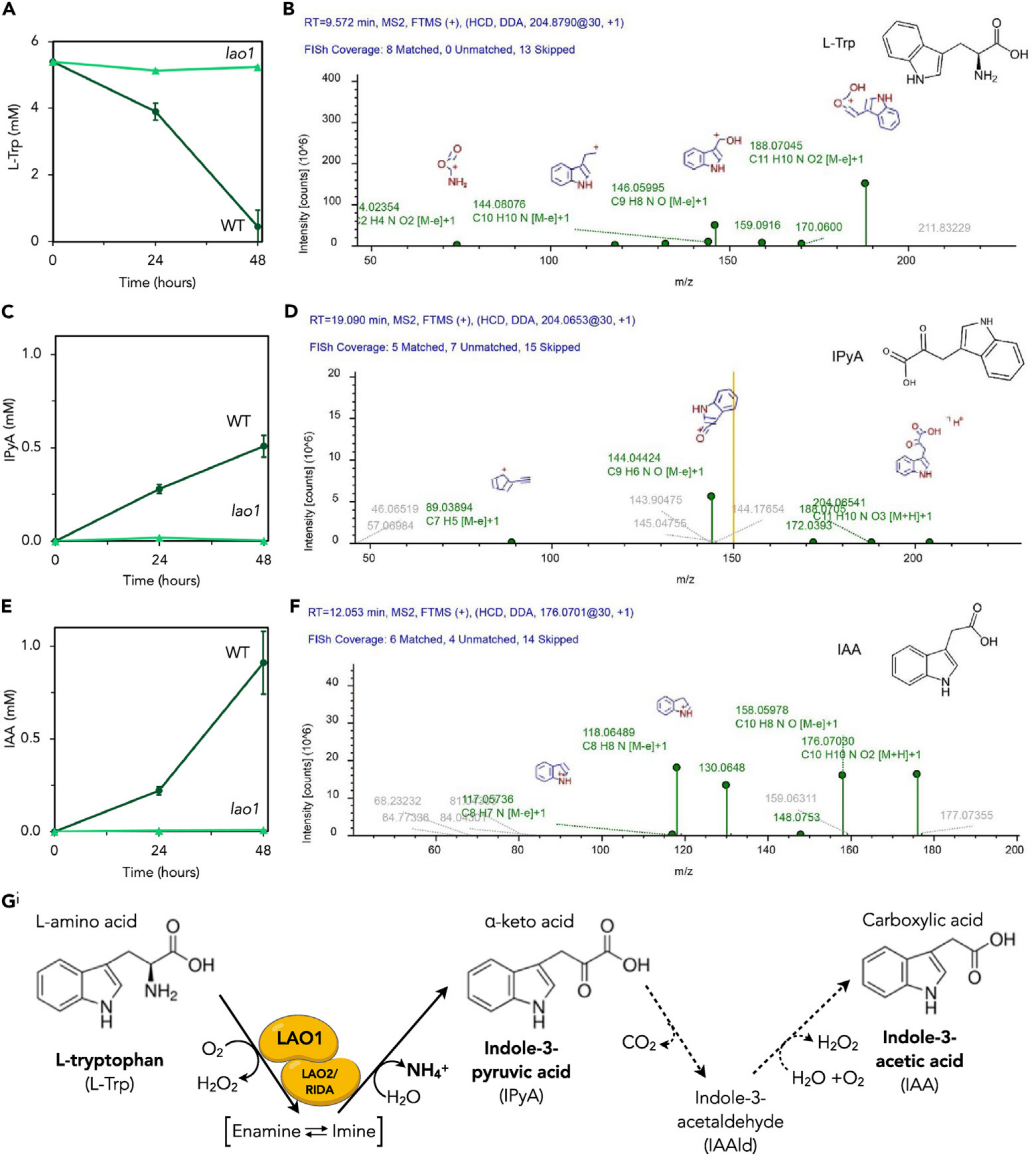
L-amino oxidase (LAO1) plays a critical role in indole-3-acetic acid (IAA) production from L-tryptophan (L-Trp) in *Chlamydomonas* *Chlamydomonas* wild-type (dark green circles) and *lao1* null mutant (light green triangles) log-phase cells at 5×10^6^ cells/ml were incubated for 48 h in nitrogen-free medium supplemented with 5 mM L-Trp as a sole source of nitrogen. This is the minimum concentration at which we observed a significant growth reduction in the absence of any other N source available (Figure S1C). (A and D) L-Trp, (B and E) indole-3-pyruvic acid (IPyA), and (C and F) IAA were quantified in the cell-free supernatants by HPLC; the identity of these compounds was confirmed with LC-MS/MS. The MS2 match was performed using the Fragment Ion Search function of Compound Discoveror 3.1 (Thermo Fisher Scientific, San Jose, USA). Green dots represent MS2 matchings for L-Trp (B), IPyA (D), and IAA (F). (*G*) In the periplasm, LAO1 and LAO2/RIDA (Reactive Intermediate/Imine Deaminase A) deaminate extracellular L-Trp to produce the α-keto acid IPyA, which is decarboxylated to IAA by means of a yet unidentified mechanism (dashed arrows).

L-tryptophan (L-Trp) is the major substrate for IAA production in bacteria, fungi, algae, and land plants (reviewed by Morffy et al.^20^). *Chlamydomonas* can transform exogenous L-Trp and other amino acids into ammonium and a corresponding α-keto acid by means of the extracellular deaminase LAO1.^39^ The *LAO1* gene is highly induced by nitrogen (N) limitation.^41^^,^^49^ This enzyme supports growth on L-Trp as the sole N source; *lao1* knockout mutants completely lose the ability to grow under these conditions (see Calatrava et al.^41^; supported by Figure S1A). To test whether *Chlamydomonas* LAO1 is involved in IAA biosynthesis, log-phase wild-type (WT) and *lao1* null mutant cells were incubated for 48 h in growth medium supplemented with 5 mM L-Trp as a sole source of N (Figure 1). The concentrations of L-Trp, the corresponding keto-acid indole-3-pyruvic acid (IPyA), and IAA were quantified in cell-free supernatants by HPLC (Figures 1A–1C, respectively), and the identity of these compounds was confirmed by LC-MS/MS (Figures 1D–1F). WT cultures depleted L-Trp and accumulated the byproducts IPyA and IAA in the medium, along with other IAA biosynthesis intermediates; indole-3-acetamide and indole-3-acetaldehyde were also present in the media but did not accumulate over time. The L-Trp metabolism product kynurenic acid also accumulated in the media (Figures S2–S5). The media of the *lao1* mutant cultures and the controls without cells did not accumulate IAA and IPyA, and the L-Trp concentration remained constant (Figures 1A and S6).

These results show that LAO1 is essential for the biosynthesis of IAA in *Chlamydomonas*. Although LAO1 mediates the deamination of L-Trp into IPyA, the first step in IAA biosynthesis, the subsequent step(s) may occur via a non-enzymatic decarboxylation of the keto acid by hydrogen peroxide^44^ or may be catalyzed by an enzyme(s) (Figure 1G). We were unsuccessful in generating definitive support for either possibility (Table S2) so the precise mechanism for IPyA conversion to IAA remains to be elucidated.

### IAA prevents *Chlamydomonas* from multiplying and attenuates chlorophyll degradation under N limitation

**Figure 2:**
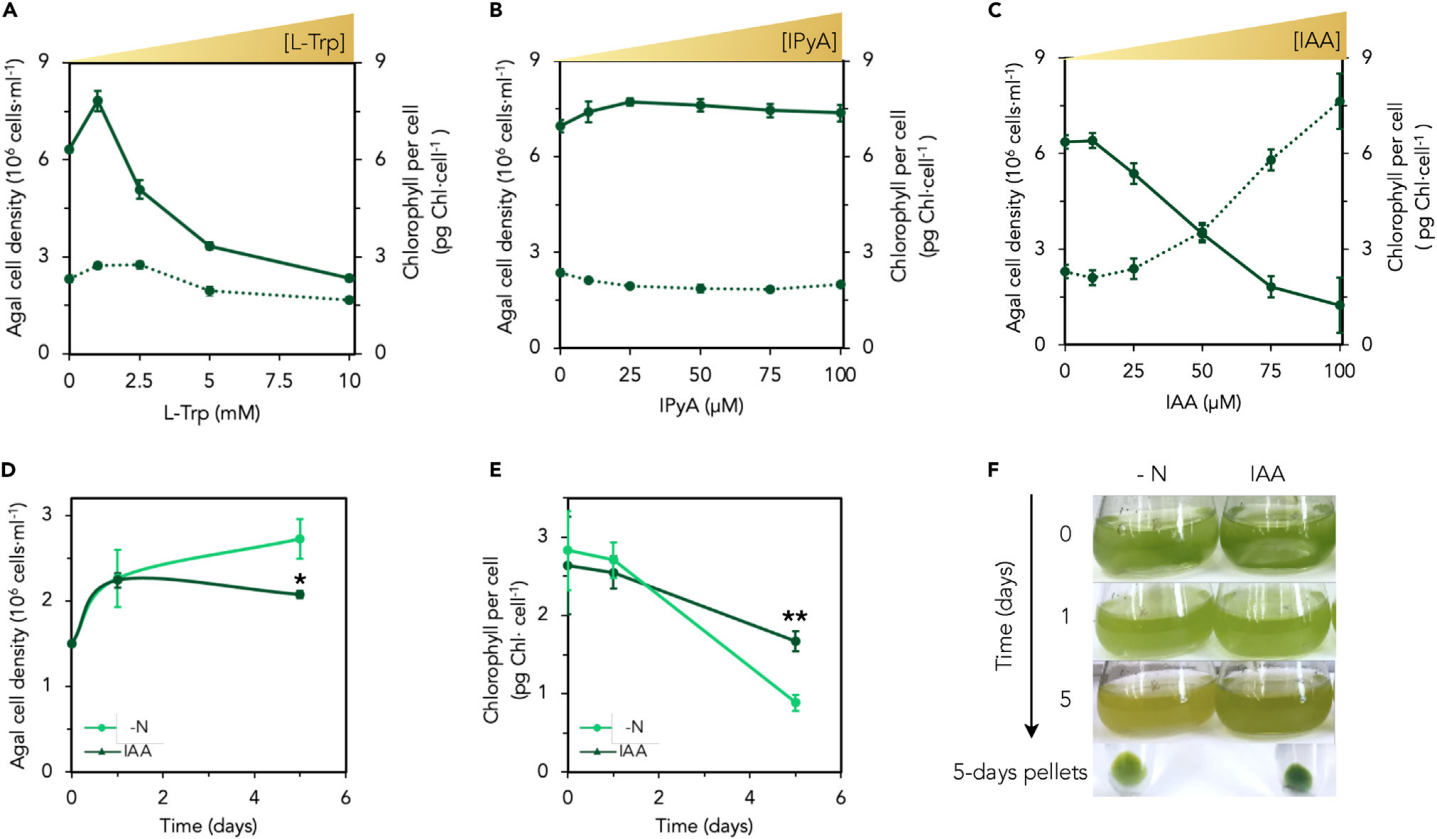
IAA arrests cell multiplication and attenuates chlorophyll degradation in nitrogen-limited *Chlamydomonas* (*A–C*) Impact of exogenously added tryptophan (L-Trp), indole-3-pyruvic acid (IPyA), and indole-3-acetic acid (IAA) on *Chlamydomonas* growth in the presence of L-alanine (L-Ala). Wild-type cells at an initial concentration of 0.2×10^6^ cells/mL were grown for three days on L-Ala (4 mM) as an N source (to enable algal growth under N-limiting conditions and ensuring LAO1 expression; see STAR Methods) in the presence of the indicated concentrations of (A) L-Trp*,* (B) IPyA, or (C) IAA. Cell culture densities are indicated as solid lines and chlorophyll content per cell as dotted lines. (D–F) Impact of IAA in *Chlamydomonas* during N deprivation. (D) Cell density and (E) chlorophyll content during N deprivation (in the absence of any assimilable N source). Wild-type cells at 10^6^ cells/ml were incubated in N-free media (–N) or supplemented with 500 μM of IAA (IAA). Data are averages of 3 biological replicates with error bars depicting standard deviations. Asterisks indicate statistically significant differences compared with the control without IAA (t test: n = 3; ∗α < 0.05; ∗∗α < 0.005; ∗∗∗α < 0.001). (F) A representative culture flask of each condition in panels (D) and (E) was imaged at the start, after one day, and after five days; cell pellets shown were harvested by centrifuging 0.5 mL of the cultures after five days of incubation.

Since IAA controls cell growth in algae,^5^^,^^7^^,^^9^^,^^10^^,^^50^ we asked whether this auxin has any effect on *Chlamydomonas* growth under the conditions for which we found IAA accumulation (i.e., under N limitation, in the absence of inorganic N). First, we tested the effect of L-Trp and LAO1 on *Chlamydomonas* growing on different N sources (Figure S1). Whereas growth on some amino acids like L-Ala and L-Trp is strictly dependent on LAO1, L-Arg can also be directly transported into the cell supporting growth independent of LAO1^41^^,^^51^ (Figure S1A). Thus, to test the impact of LAO1, we compared the growth of WT and *lao1* strains growing on media with L-Arg, supplemented with a range of L-Trp concentrations (Figure S1B). We observed that for WT cells, growth was improved at lower L-Trp concentrations up to 5 mM. However, this improvement was not observable at higher concentrations (10 mM) (Figure S1B). Growth of the *lao1* mutant was not affected by L-Trp supplementation, however, suggesting that this concentration-dependent effect of L-Trp is due to LAO1 activity. We also tested the effect of L-Trp addition on cells growing on L-Ala as another potential LAO1-dependent N source; in this case, we only tested this using the WT strain because the *lao1* mutant cannot grow on L-Ala (or L-Trp) (Figure S1A). We observed that higher concentrations of L-Trp inhibited growth, regardless of whether they were grown on L-Ala (Figure 2A) or not (Figure S1C), suggesting that the growth effects were L-Trp-specific.

Lastly, we examined the impact of L-Trp addition on WT and *lao1* cells growing on ammonium, which can support growth independently of LAO1 and is also a strong repressor of LAO1 expression.^41^ In contrast to the phenotype observed in cells growing on L-Ala, no impact of L-Trp addition was observed for WT cells when grown on ammonium (Figure S1D). This result can be explained by the absence of LAO1 in ammonium and is consistent with the role of this enzyme in the concentration-dependent effect of L-Trp that we observed with L-Ala. To better understand the effect of LAO1-produced IAA on *Chlamydomonas* growth, cells were supplemented with different concentrations of L-Trp, IPyA, and IAA; this was conducted in the presence of L-Ala to ensure basal growth and that LAO1 is expressed (Figures 2A–2C). Note that given the presence of L-Ala, we did not expect the rate of IPyA/IAA accumulation in this experiment to be as high as the results shown in Figure 1 because LAO1 has a higher specific activity for L-Ala than for L-Trp.^40^ We initially tried a wider range of IAA concentrations (not shown) but chose to use a limited range of concentrations of IAA: from the minimum concentration that does not affect growth (12.5 μM IAA) to a maximum that reaches a level similar to that for 10 mM L-Trp (100 μM IAA). Similar concentrations of IPyA were used as with IAA to enable fair comparisons with IAA results. A slight increase in cell yield was observed at 1 mM L-Trp (Figure 2A). This could be explained by a slightly higher total N input and/or due to the production of IAA, which could have a stimulatory effect at low concentrations.^50^ Growth reduction was observed at higher concentrations of L-Trp, however, which could be attributed to the accumulation of IAA and other byproducts. Indeed, IAA concentrations higher than 10 μM had a clear inhibitory effect on culture cell density, with almost 70% inhibition at 100 μM IAA (Figure 2C). In contrast, similar levels of the intermediate IPyA did not affect growth (Figure 2B). This is consistent with the inability we observed of producing IAA directly from IPyA under these conditions (Table S2), but this may be due to the low stability of IPyA in solution^52^^,^^53^ so these results must be interpreted cautiously.

The reduced cell density observed with increasing concentrations of extracellular IAA was correlated with an increase (up to four times) in chlorophyll content per cell (Figure 2C), which was not observed with IPyA (Figure 2B). This effect was also not observed with high levels of L-Trp (Figure 2A), possibly because additional intermediates produced from L-Trp deamination (e.g., hydrogen peroxide) may impair the accumulation of chlorophyll. N limitation generally leads to chlorophyll degradation,^49^ so the higher chlorophyll content per cell we observed in response to exogenous IAA could be explained by a reduction in chlorophyll degradation, or an increase in cell volume or the formation of palmelloids,^54^^,^^55^^,^^56^ which must be IAA-specific and not merely LAO1-specific. To test this, we incubated concentrated, N-starved *Chlamydomonas* cultures with IAA and indeed found that the degradation of chlorophyll during N deprivation was reduced (Figures 2D–2F and S7) with no apparent effect on cell volume and/or palmelloid formation. In *Chlamydomonas*, chlorophyll degradation under N deprivation is linked to the mobilization of stored N to allow cells to duplicate one additional round before the complete cessation of growth.^49^ Here, we observed that despite being N-deprived, *Chlamydomonas* cultures significantly reduce cell multiplication rate in the presence of IAA, which may explain the increased chlorophyll content of these cells.

### IAA production in *Chlamydomonas* facilitates a mutualistic interaction with *Methylobacterium aquaticum*

**Figure 3:**
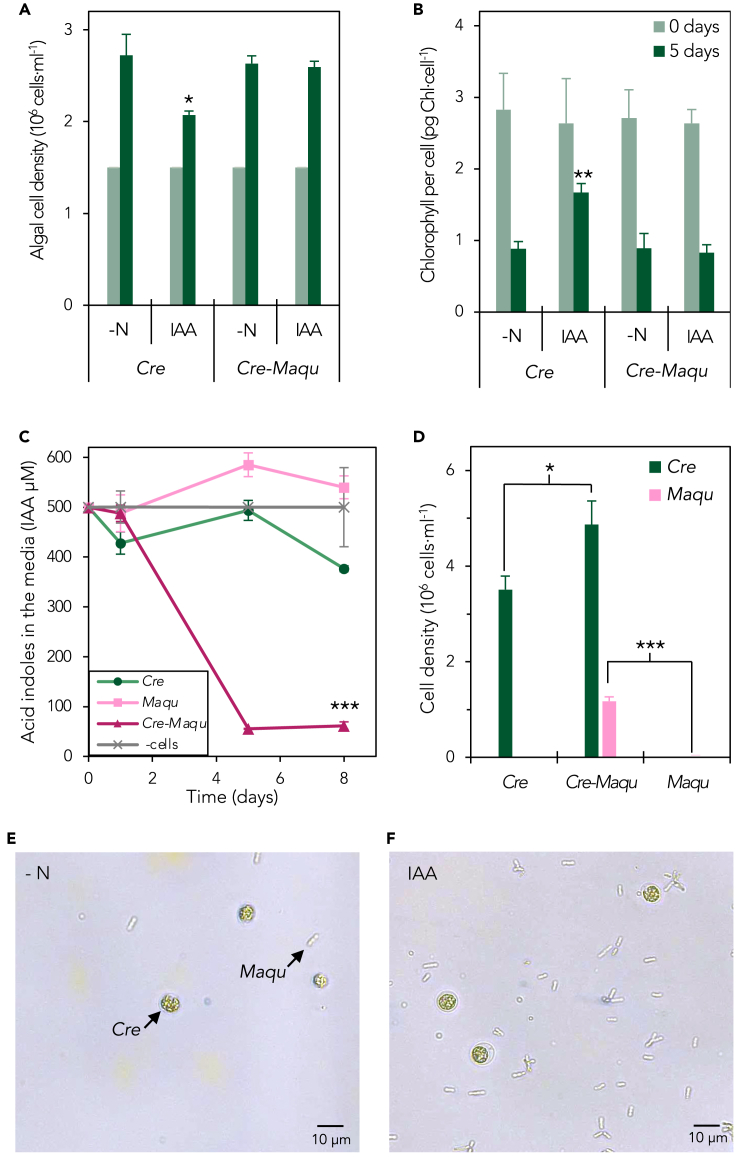
*Methylobacterium aquaticum* reduces IAA levels in *Chlamydomonas* cultures to relieving algal inhibition of cell multiplication and chlorophyll degradation (A) Algal cell density and (B) chlorophyll content per cell were determined initially (0 days) and after five days of incubation for *Chlamydomonas* monocultures (Cre) and *Chlamydomonas-M. aquaticum* co-cultures (Cre-Maqu) in N-free medium (–N) supplemented with 500 μM IAA (IAA). Initial cell concentrations were 10^6^ cells/ml for *Chlamydomonas* and A_600_ of 0.01 for *M. aquaticum* (approximately 10^6^ cells/mL). *Chlamydomonas* monocultures correspond to the same dataset as in Figures 2D and 2E and are represented here as a reference for algal monocultures. (C) *Chlamydomonas* (Cre) monocultures, *M. aquaticum* (Maqu) monocultures, and co-cultures (Cre-Maqu*)* were incubated on N-free media supplemented with 500 μM of IAA for five days. Indoles concentration in the cell-free media were determined using the Salkowski reagent (see STAR Methods). (D) Algal and bacterial cell densities after eight days of growth on 500 μM of IAA in coculture or monoculture were quantified using qPCR of single-copy genes specific for *Chlamydomonas* (*centrin*) or *M. aquaticum* (*rpoB*) (see STAR Methods). Data shown are averages of 3 biological replicates with error bars depicting standard deviations. Asterisks indicate statistically significant differences compared with the control comparison (t test: n = 3; ∗α < 0.05; ∗∗α < 0.005; ∗∗∗α < 0.001). (E) *Chlamydomonas-M. aquaticum* cocultures were imaged using a light microscope under conditions without a nitrogen source (-N) and (*F*) supplemented with 500 μM IAA (IAA) after five days.

Because IAA mediates plant/algal-bacterial interactions, we asked whether IAA and LAO1 could play a role in the establishment of interactions of *Chlamydomonas* with the bacterial genus of *Methylobacterium*, given prior work demonstrating mutualistic tendencies of this taxa with *Chlamydomonas* under N-limiting conditions.^37^ Following-up on this work, we quantified the growth of *Chlamydomonas* in coculture with 10 different species of methylobacteria using L-Trp as the sole N source. We observed that coculturing with *Methylobacterium* spp., including *M. aquaticum*, improved algal growth (Figure S8). Naively, this growth promotion could simply be due to methylobacteria mineralizing L-Trp to provision *Chlamydomonas* with ammonium, which is more efficiently used by this alga than L-Trp as we similarly reported for L-proline.^37^ However, this seems unlikely because we observed no algal growth promotion of the *lao1* knockout mutant by methylobacteria (Figure S8), suggesting that LAO1 is essential for this growth enhancement. Given the conversion of L-Trp to IAA and the inhibitory effects of IAA on *Chlamydomonas*, we hypothesized that methylobacteria may promote algal cell growth by reducing the levels of IAA accumulated in the media in coculture relative to *Chlamydomonas* in monoculture. Indeed, in coculture with *M. aquaticum*, there was no inhibition of growth by IAA and no effect on chlorophyll content (Figures 3B and 3C), and measured auxin levels were highly reduced (Figure 3D), strongly supporting a methylobacteria-mediated degradation of IAA. Because auxin concentrations were not reduced in bacterial monocultures nor in algal monocultures, both microbes are necessary for auxin to be eliminated from the media in a cooperative fashion. This effect was tested with nine other *Methylobacterium* spp. but was only observed with *M. aquaticum*, showing a species-specific interaction (cf. Figure S9)*.* Importantly, coculturing with exogenously added IAA resulted in higher cell density for both microbes compared with the respective monocultures (Figures 3E–3G). Therefore, both microbes mutually benefited from the presence of each other. In this medium, acetate can serve as a source of carbon and energy for both the alga and the bacterium.^42^^,^^57^ However, no N source other than 500 μM IAA was added to the media to ensure N limitation, thus no large growth rates were expected. Because no detectable ammonium was produced by the bacterial monocultures on IAA (Table S3), *Chlamydomonas* most likely benefits from relief of IAA-induced inhibition through the bacterial degradation of this auxin. On the other hand, this observation strongly suggests that the bacterium, prompted by the presence of the alga, is feeding on IAA as an N source, allowing it to thrive in coculture.

## Discussion

The interactions between algae and heterotrophic bacteria play a fundamental ecological role controlling nutrient cycling and biomass production in their habitats.^58^ Studies have shown that bacteria can produce the phytohormone IAA to promote algal growth,^5^ resembling the role of this molecule in plant-bacterial associations. However, auxin production by the algal partner has been largely overlooked or neglected,^5^^,^^12^ despite the important ecological and economic impact this may cause in both aquatic and terrestrial ecosystems.^59^^,^^60^ Moreover, there has been no known genetic pathway to produce IAA in algae thus far. Here, we show a novel and unexpected mechanism of IAA production in the model alga *Chlamydomonas* under N-limiting conditions. *Chlamydomonas* can deaminate L-Trp extracellularly via the LAAO enzyme LAO1 to yield the keto acid IPyA, the major precursor for auxin biosynthesis in plants, bacteria, and fungi.^20^ A pathway for tryptophan-dependent IAA production involving an LAAO gene was recently discovered in the plant-benefiting bacterium *Gluconacetobacter diazotrophicus* encoded by a gene cluster containing L-amino acid oxidase, cytochrome *c*, and *ridA* genes.^47^ *Chlamydomonas* harbors a similar gene cluster that includes L-amino acid oxidase (*LAO1*) and a putative *RidA* gene (*LAO2*). We recently characterized a *Chlamydomonas lao1* knockout mutant that lacks extracellular LAAO activity that we used here to evaluate the relevance of this enzyme for IAA production.^41^ We have shown that LAO1 is essential to produce IAA from L-Trp via an IPyA intermediate. Whereas this enzyme is most likely involved in the initial step of L-Trp deamination to yield IPyA, the conversion of IPyA into IAA may be achieved by a two-step pathway mediated by ipdC decarboxylase (via indole-3-acetaldehyde intermediate) in some bacteria or by the single-step YUCCA pathway (flavin monooxygenase-like enzyme) in plants.^20^ As in *G. diazotrophicus*, no homologs for any of these enzymes have been identified yet in *Chlamydomonas*, and thus, the nature of the conversion of IPyA into IAA in both organisms awaits experimental confirmation. This mechanism could involve a non-enzymatic decarboxylation of the keto acid by hydrogen peroxide, although our results do not support this. Alternatively, it may require an enzyme that relies on the presence of L-Trp and/or LAO1 to be expressed, as the addition of IPyA did not yield IAA in either of the two studies. However, this may be due to the low stability of IPyA in solution so these results must be interpreted cautiously. In addition to IPyA and IAA, kynurenic acid was found to accumulate in the algal media. This L-Trp metabolite can be produced via IPyA oxidation by free radicals.^61^^,^^62^ Although the role of kynurenic acid in algae remains largely unexplored, a few reports have shown that it is produced by algae and cyanobacteria and, interestingly, is implicated in mutualistic interactions involving bacteria.^63^^,^^64^^,^^65^ Here, we demonstrated a LAO1- and L-Trp-dependent mutualism between *Chlamydomonas* and *M. aquaticum* mediated by IAA, although other L-Trp metabolites like kynurenic acid may be responsible for mutualism with other *Methylobacterium* species and bacteria. Whether kynurenic acid production by *Chlamydomonas* is involved in mutualistic interactions with other microbes is unknown, but we believe this merits further investigation.

Previously limited to a single bacterial species, the role of LAAO enzymes in IAA production has been broadened here to include the microalga *Chlamydomonas* and may extend to other algae that harbor LAO1 homologs including members of Rhodophyta, Alveolata, Haptophyta, Heterokonta and Dinophyta.^41^ In support of this idea, the haptophyte alga *Emiliania huxleyi* harbors two putative *LAO1* gene homologs^41^ and shows a ratio of L-Trp conversion to IAA similar to what we found here in *Chlamydomonas* (16%–20% of L-Trp conversion).^17^ However, based on existing genomic data, the *LAO1* gene seems to be absent in most members of the green lineage (i.e., green algae and land plants) other than *Chlamydomonas* spp., so they must rely on alternative pathways for IAA biosynthesis.^41^ Higher levels for IAA production in *Chlamydomonas* have been reported relative to 23 other green algal species,^66^ consistent with *Chlamydomonas* producing IAA using a different strategy compared with sister lineages. To date, transcriptomic and/or bioinformatic analyses appear to indicate that the most common putative pathway for IAA biosynthesis in microalgae, including *Chlamydomonas*, occurs via an indole-3-acetamide intermediate (reviewed by De Smet et al.^67^). Although we have shown that LAO1 is crucial for IAA production under N limitation, alternative pathways that remain to be elucidated may be relevant under different conditions.

The *LAO1* gene in *Chlamydomonas* was likely acquired through horizontal gene transfer, suggesting an adaptive advantage of having *LAO1* in *Chlamydomonas’* native environment.^41^ Originally isolated from soil, this alga has been readily found in the rhizosphere,^68^ where inorganic N may be limiting but free amino acids may be present from plant exudates and microbial decay.^69^^,^^70^^,^^71^ In this context, LAO1 could play a prominent role in scavenging N from a broad range of amino acids.^41^ As a periplasmically targeted enzyme in *Chlamydomonas*,^39^^,^^40^ LAO1-driven deamination of amino acids may release ammonium for its own use, and keto acids could potentially act as chemoattractants and/or a diffusible “public good” for closely associated or nearby partners.^72^^,^^73^^,^^74^ The accumulation of LAO1 keto acid byproducts as public goods in the phycosphere that are unused by the alga could have significant ecological roles that have been thus far neglected. Here, we have shown one example of how LAO1 can lead to extracellular IAA production with physiological and ecological consequences for the alga. Although relatively low concentrations of IAA can improve algal growth,^50^ we observed that the accumulation of high levels of IAA in the media arrests algal cell proliferation and chlorophyll degradation. This is consistent with previous observations of higher chlorophyll accumulation in response to IAA in the microalgae *Chlorella vulgaris*, *Nannochloropsis oculata*, and *Acutodesmus obliquus*.^75^^,^^76^^,^^77^ An increase in photosynthetic performance by IAA has also been observed in the microalgae *E. huxleyi* and diatoms that harbor IAA-producing symbiotic bacteria.^5^^,^^17^ Similar effects have been observed in the chloroplasts of plants.^78^ This effect of IAA on the chlorophyll content is stronger in aged algal cultures and plant samples^17^^,^^78^^,^^79^ and might be linked to a condition of stress.^80^ We considered the effect of IAA on chlorophyll levels in the context of N limitation as a stress condition that triggers LAO1 accumulation. Under mixotrophic conditions (i.e., in the presence of acetate), this alga prioritizes cellular respiration over photosynthesis.^49^ As a result, when N is limiting, pigments and other N-containing macromolecules involved in photosynthesis and chloroplast function are degraded first, presumably to enable *Chlamydomonas* cells to undergo an additional round of duplication.^49^ The accumulation of exogenous IAA by *Chlamydomonas* could function as a quorum-sensing-like signal molecule to decrease growth when the cell population is high and nutrient resources are limited.^12^^,^^18^ Preventing cell multiplication while avoiding the breakdown of the photosynthetic machinery during N limitation may be a hedge-betting strategy to capitalize on mutualistic cross-feeding interactions with other N-mineralizing microbes in the vicinity (e.g., *Methylobacterium*). These neighboring microbes may benefit from the provisioning of photosynthates like glycerol in exchange for more readily assimilable sources of N like ammonium that *Chlamydomonas* can use to resume growth.^37^^,^^81^

IAA can be metabolized by rhizospheric bacteria,^82^^,^^83^^,^^84^^,^^85^ some of which like *Pseudomonas putida* show chemotaxis toward the auxin.^85^^,^^86^ Thus, IAA production by *Chlamydomonas* may attract and feed bacteria that could potentially be beneficially self-serving. When amino acids are present but inorganic N is absent, *Chlamydomonas* interacts mutualistically via C and N cross-feeding with *Methylobacterium* spp., species that have been shown to cooccur with *Chlamydomonas* and that are part of the phycosphere of other green algae.^87^^,^^88^^,^^89^ Here, we demonstrate another mutualistic mode of interaction between *Chlamydomonas* and a *Methylobacterium* sp., mediated through IAA. *M. aquaticum*, induced or complemented by the presence of *Chlamydomonas*, can feed on IAA, which can “release the brake” on *Chlamydomonas* growth and facilitate the enhanced growth of both organisms. From the algal perspective, LAO1-mediated production of extracellular ammonium and keto acids may thus constitute a multifaceted strategy of (1) signaling, (2) waiting for partner feedback, (3) followed by mutualistic stimulation that may mirror a sort of call-and-response dynamic of plant-microbe interactions.^90^

To our knowledge, this is the first study to report an algal-dependent mechanism for IAA degradation in bacteria and IAA degradation by *Methylobacterium* spp. in general. Bacterial genes for IAA catabolism (*iac*)^91^ appear to be absent from available *Methylobacterium* genomes but we believe that IAA degradation by *M. aquaticum* (involving a yet unidentified pathway) may cause a carbon/nitrogen metabolic imbalance that hampers its growth in the absence of the alga.^92^ During coculture, algal-derived photosynthates may restore carbon/nitrogen balance and enhance bacterial growth.^37^ We cannot rule out the possibility that other putative metabolites or proteins produced by the alga may also potentially facilitate bacterial degradation of IAA. Regardless, this observation supports that IAA-degrading bacteria can influence not only land plants but also algae.^91^ A previous study found that the haptophyte microalga *E. huxleyi* exhibits strain-specific differences in the production of IAA and susceptibility to infection by the pathogenic roseobacter *Ruegeria* sp. R11: while the non-IAA producer strain is resistant to roseobacter, the IAA-producing strain is susceptible to bacterial infection.^17^ This bacterial infection is accelerated by the addition of L-Trp to the coculture, which suggests that L-Trp-derived IAA production by the alga could enhance bacterial infection. However, because the levels of IAA in the algal cultures were shown to be reduced in coculture with roseobacter, the role of algal-derived IAA production was not pursued.^17^ Given the findings presented here showing that bacterial degradation of IAA can be triggered in the presence of algae, we speculate that in the presence of *E. huxleyi*, *Ruegeria* sp. may metabolize algal-derived IAA to achieve higher bacterial densities and potentially accelerate algal infection. Regardless, we believe that algal-dependent bacterial degradation of IAA may be relevant for many other bacterial species and their interactions with both algae and land plants.

**Figure 4:**
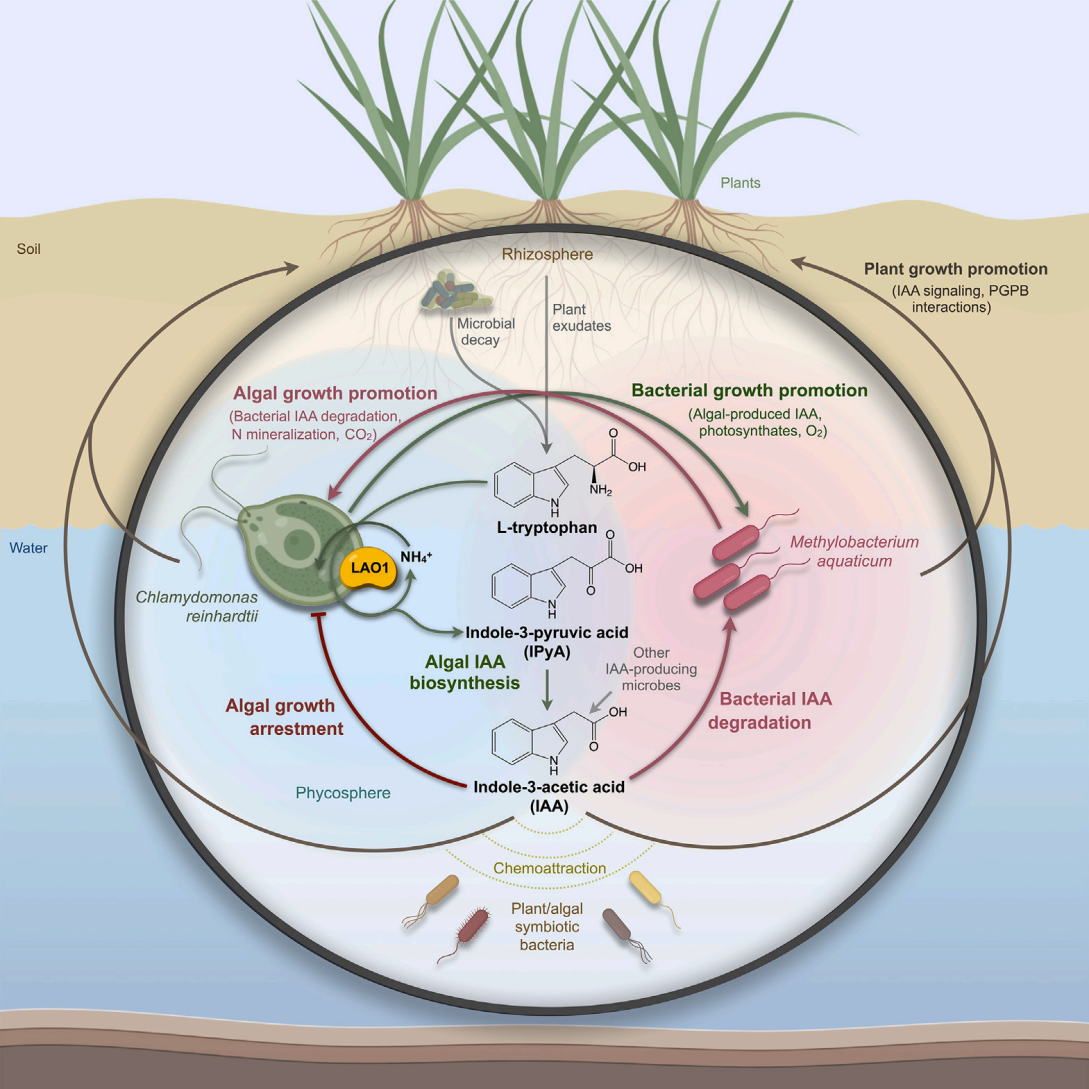
Proposed role of auxin-mediated mutualistic interactions between *Chlamydomonas*, *Methylobacterium*, and land plants For a Figure360 author presentation of this Figure 4, see https://doi.org/10.1016/j.isci.2023.108762. In soil environments where tryptophan may be present due to plant exudation and microbial decay, bacterial indole-3-acetic acid (IAA) biosynthesis from this amino acid can promote plant growth. *Chlamydomonas reinhardtii* can also convert tryptophan into IAA using the extracellular enzyme LAO1. Accumulation of this auxin may result in algal growth arrest and in attracting beneficial PGPB bacteria. In the presence of the IAA-degrading bacterium *Methylobacterium aquaticum*, IAA is depleted, enhancing growth of both microorganisms. Metabolites exchanged between bacteria and algae could strengthen this mutualistic interaction.^37^ Moreover, we imagine three-way algal-plant-bacterial associations whereby algal-derived IAA not only benefits the alga but promotes plant-bacterial symbioses and modulates plant physiology directly. Figure created using BioRender.com and ChemDraw 20.1. [∗] indicates interactions or processes shown in this work. Numbers in square brackets correspond to references: [1] Vallon et al.^40^; [2] Cox et al.^1^^,^^4^^,^^8^; [3] Rico-Jiménez et al.^85^; [4] Kravchenko et al.^70^; [5] Moe LA^108^; [6] Backer et al.^109^

Among the 10 *Methylobacterium* spp. tested in this work, *M. aquaticum* was significantly more efficient in degrading IAA in the presence of *Chlamydomonas*. This may underlie the basis for this particular bacterial species’ broad ability to enhance the growth of early diverging plant lineages like green algae and moss.^37^^,^^93^ *Chlamydomonas* and *M. aquaticum* have both been found in freshwater environments and in the rhizosphere.^94^^,^^95^^,^^96^ Given niche overlap of algae and higher plants in soil and the ability of algae to “dialogue” with other microbes using the same auxin compounds that regulate land plants growth and development, we hypothesize that algae may play an underappreciated role in the recruitment of PGPB to the plant microbiome, let alone in modulating plant fitness through algal-plant interactions (Figure 4). Plant-plant interactions dramatically shape plant fitness, coexistence, life histories, and community assembly^36^^,^^97^; we believe it is likely that the interactions of plants with earlier-diverging green lineages are also important and relevant to contemporary ecosystems.^98^^,^^99^

The role of microalgae as players in the plant microbiome has only recently started to be appreciated and their use to improve soil fertility, water preservation, and plant growth is now emerging as a promising approach for sustainable agriculture^48^^,^^59^^,^^100^^,^^101^^,^^102^^,^^103^^,^^104^ (see Alvarez et al.^105^ for a recent review). For these new applications, understanding how and under what environmental conditions IAA is produced by rhizospheric and phyllospheric algae is of great interest. Bacterial degradation of IAA has been shown to be essential for rhizosphere colonization and plant growth promotion,^106^ and similar to how LAAO-mediated auxin production is involved in plant growth promotion by *G. diazotrophicus*,^47^ auxin production by other LAAO-containing microbes in the rhizosphere like *Chlamydomonas* could have an impact on plant growth and fitness. Our findings suggest that future studies should carefully consider the role that terrestrial algae play in plant microbiomes^48^ and in the historical record of land plant evolution.^107^

### Conclusions

We provide genetic evidence that supports IAA production by the model alga *Chlamydomonas* via an unexpected LAAO-mediated pathway, which has thus far been restricted to a single bacterial species.^47^ This finding supports a new role for microbial LAAOs in auxin production that may be widespread in other algal lineages.^17^^,^^41^ We also show that IAA may act as an extracellular self-regulatory/quorum-sensing-like molecule to control cell multiplication and delay the breakdown of the photosynthetic machinery under inorganic N-limitation. This may be an ecological strategy to facilitate interactions with N-mineralizing bacteria. We demonstrate that the plant- and algal-benefiting bacteria *Methylobacterium* spp. can degrade extracellular levels of IAA generated by the alga in the presence of L-Trp and N limitation to alleviate inhibition of algal cell multiplication. Interestingly, these bacteria can feed on IAA only in the presence of the alga, revealing a new cooperative mode of auxin degradation that is induced and/or functionally complemented by the alga. This new mode suggests a role for algal-driven IAA production prompted by algal-bacterial interactions that may be relevant to plant-microbiome dynamics. Because *Chlamydomonas* and *Methylobacterium* are naturally found in the rhizosphere, their role in modulating IAA levels may impact plant fitness and could be exploited for crop improvement in sustainable agriculture. Overall, this work extends our understanding of auxin production and degradation by algae and algal-bacterial consortia under N-limitation, highlighting the potential for tri-partite interactions between rhizospheric algae, PGPB, and land plants.

### Limitations of the study

The results of this study show that LAO1 is essential for the biosynthesis of IAA in *Chlamydomonas*. Although LAO1 is involved in the first step of IAA biosynthesis, namely L-Trp deamination, the subsequent step(s) for IPyA conversion to IAA remains unclear. Whereas algal growth on L-Trp was improved by four different *Methylobacterium* spp., IAA degradation could be attributed only to *M. aquaticum* in this work. Additional analyses of the metabolites from algal-bacterial cocultures using other *Methylobacterium* species would be beneficial in revealing the basis of other potential interactions.

## STAR★Methods

### Key resources table

**Table:** 

REAGENT or RESOURCE	SOURCE	IDENTIFIER
**Experimental models: Organisms/strains**		

*Chlamydomonas reinhardtii* wild-type strain	CLiP, Chlamydomonas Resource Center, University of Minnesota	CC-5325
*Chlamydomonas reinhardtii lao1* mutant	CLiP, Chlamydomonas Resource Center, University of Minnesota; Calatrava et al.^41^	LMJ.RY0402.044073
*Methylobacterium oryzae* strain CBMB20	DSMZ	18207 (DSMZ)
*Methylobacterium* sp. strain 88A	Kindly provided by Prof. Ludmila Chistoserdova	1131813 (NCBI)
*Methylobacterium organophilum* strain XX	BCCM/LMG	ATCC 27886
*Methylobacterium* sp. strain M017	Calatrava et al.^37^	MG287145.1 (Genebank)
*Methylobacterium marchantiae* strain JT1	DSMZ	21328 (DSMZ)
*Methylobacterium hispanicum* strain GP34	CECT	16371 (CECT)
*Methylobacterium nodulans*	BCCM/LMG	21967 (BCCM/LMG)
*Methylobacterium aquaticum* strain GR16T	DSMZ	16371 (DSMZ)
*Methylobacterium aerolatum* strain 5413S-11	DSMZ	19013 (DSMZ)
*Methylorubrum* (formerly *Methylobacterium*) *extorquens* strain AM1	Kindly provided by Prof. Cecilia Martinez-Gomez	9133 (NCBI)

**Deposited data**		

The original data generated in this work has been deposited in Mendeley Data	This work	Mendeley Data: https://doi.org/10.17632/bf5d3hn32z.1

**Oligonucleotides**		

Cen1CreU (5’-TTACAAGATGGGACAGCCCG-3’)	Calatrava et al.^37^	N/A
Cen1CreL (5’-CAGCCCGCAGAGGAACTAAC-3’)	Calatrava et al.^37^	N/A
rpoBMaqU (5’-TAGATGTAGCCGACCGTGAC-3’)	Calatrava et al.^37^	N/A
rpoBMaqL (5’- ATGAAGGCGATCTACAGCGA-3’)	Calatrava et al.^37^	N/A

### Resource availability

#### Lead contact

Further information and requests for resources and reagents should be directed to and will be fulfilled by the lead contact, Victoria Calatrava (b82capom@uco.es).

#### Materials availability

This study did not generate new unique reagents.

#### Data and code availability

• Original data have been deposited at Mendeley Data (https://data.mendeley.com/) and are publicly available as of the date of publication. Accession number is listed in the key resources table.
• This paper does not report original code.
• Any additional information required to reanalyze the data reported in this paper is available from the lead contact upon request.

### Experimental model and study participant details

*Chlamydomonas reinhardtii* wild-type strain CC-5325 and *lao1* mutant LMJ.RY0402.044073 were obtained from the *Chlamydomonas Resource Center* (https://www.chlamycollection.org/). This *lao1* mutant contains a CIB gene cassette insertion at the *Cre12.g551352* locus, corresponding to the *LAO1* gene,^110^ that prevents LAO1 function.^41^ Cells were pre-cultured for 2-3 days in Tris-Acetate-Phosphate (TAP) medium^111^ containing 8 mM of ammonium chloride at 23°C under continuous light and agitation (120 rpm).

*Methylobacterium* strains used in this work are summarized in the key resources table. Bacterial cells were pre-cultured in *Methylobacterium* Medium (MeM)^37^ supplemented with 2.5 g·l^-1^ of tryptone (MeM^T^) for 2-3 days at 28°C and continuous agitation (150 rpm).

To detect potential contamination, cell inocula were routinely checked by streaking on agar plates of TAP supplemented with yeast extract (2.5 g·l^-1^), incubated for 2 weeks at 23°C and examined under a light microscope.

### Method details

#### Determination of L-tryptophan metabolization, and indole-3-pyruvic acid and indole-3-acetic acid biosynthesis by Chlamydomonas using HPLC

*Chlamydomonas* wild-type and *lao1* mutant were pre-cultured as described above to exponential phase. Cells were harvested by centrifugation for 2-3 min at 2,090 x *g* and washed three times with nitrogen-free TAP medium (T-N). Cells were subsequently incubated in T-N medium supplemented with 5 mM of L-Trp under continuous light and agitation (120 rpm). After 24 and 48 hours, cell-free supernatants were stored at -20°C until analyzed. L-Trp, indole-3-pyruvic acid and indole-3-acetic acid analysis was performed by the Chromatography Department staff at the Central Service for Research Support (SCAI) of the University of Córdoba, using High-Performance Liquid Chromatography (HPLC) and UV-Vis detection. A mixture of L-Trp (Sigma-Aldrich, Spain), indole-3-pyruvic acid (Sigma, Spain) and indole-3-acetic acid (GoldBio, US) was included as standard.

#### LC-MS/MS analysis

*Chlamydomonas* wild-type cells were pre-cultured and incubated as described above in TAP-N medium supplemented with 5 mM of L-Trp under continuous light and agitation (120 rpm). After 24 and 48 and 96 hours, 300 μl of cell-free supernatants were stored at -20°C until analyzed. Freeze-dried extracts were resuspended in 2 ml of ultrapure water (Milli-Q A10 Advantage, MilliporeSigma, Burlington, MA) and applied to a Fisherbrand PrepSep C18 solid-phase extraction (SPE) column (FisherScientific, Pittsburgh, PA) that was pre-treated with 2 ml 100% HPLC grade methanol (A452-4, Fisher Scientific, Fair Lawn, NJ) followed by 2 ml ultrapure water and then 2 ml of 1 M HPLC grade acetic acid (AX0074-6, EMD Chemicals Inc., Gibbstown, NJ). The column was washed with 1 ml of 1 M acetic acid followed by 1 ml of 1% acetic acid. Analytes were then eluted using 2 ml of 100% methanol and evaporated until dried. UHPLC-MS/MS analysis of purified extracts was performed on an Orbitrap Exploris 240 instrument (Thermo Scientific, San Jose, CA) coupled to a Dionex Ultimate 3000 UHPLC system. Samples were loaded onto a PepMap 100 C18 column (0.3 mm × 150 mm, 2 μm, Thermo Fisher Scientific). Separation of the samples was performed using mobile phase A (0.1% formic acid in water) and mobile phase B (0.1% formic acid in acetonitrile) at a rate of 5 μl/min. The samples were eluted with a gradient consisting of 2% to 60% solvent B over 13 min, ramped to 95% B over 2 min, held for 8 min, and then returned to 2% B over 1 min and held for 8 min. All data were acquired in positive ion mode. Collision induced dissociation (CID) was used to fragment molecules, with an isolation width of 2 m/z units. The spray voltage was set to 3900 V, and the temperature of the heated capillary was set to 300°C. In CID mode, full MS scans were acquired from m/z 150 to 1800 followed by eight subsequent MS^2^ scans on the top ten most abundant peaks. The orbitrap resolution for the MS1 scan was 60,000 and MS2 scans was 30,000. The expected mass accuracy based on external calibration was <3 ppm. Compound Discoverer 3.1 (Thermo Fisher Scientific, San Jose, USA) with the mzCloud database was used for metabolite identification, with mass tolerance set to 0.05 Da and retention time tolerance set to 0.2 min. A Fragment Ion Search (FISh) was subsequently performed on the compounds annotated by mzCloud. Compounds exhibiting a FISh score higher than 60 were considered as successfully identified compounds. Raw UHPLC-MS/MS files for these data have been deposited in the MassIVE database (https://massive.ucsd.edu/ProteoSAFe/static/massive.jsp) with accession number MSV000092315.

#### Chlamydomonas cell growth tests

To test *Chlamydomonas* growth, cells were pre-cultured and washed as described above, placed in wells of a 48-well culture plate (BRANDplates, BrandTech Scientific, US) and incubated in fresh culture medium at an initial concentration of 0.2x10^6^ cells/ml. In these growth assays L-alanine was supplemented in the medium as indicated in the figure captions to allow algal growth while ensuring N-limiting conditions and LAO1 activity. At the indicated times, cell concentrations were determined using 100-200 μl in a microcell counter (Sysmex F-500, Sysmed Inc, Europe).

#### Chlorophyll determination

Chlorophyll (a+b) was extracted from 1 ml of algal or algal-bacterial pellets with ethanol (1 ml) and measured spectrophotometrically (Beckman Coulter, DU 800). Total chlorophyll concentration in the cultures (μg/ml) was calculated according to Wintermans and de Mots^112^: (6.1 · A_665_) + (20.04 · A_649_).

#### Indole-3-acetic acid degradation tests

To determine microbial indole-3-acetic acid depletion from the medium, *Chlamydomonas* and *Methylobacterium* spp. mono- and co-cultures were initially pre-cultured independently as described above. After 2 days (exponential growth phase), cells were washed using T-N medium and incubated in fresh T-N medium supplemented with 500 μM of IAA under continuous light and agitation (120 rpm). Initial cell densities if algae and bacteria were set at 1.5x10^6^ cells/ml and A_600_=0.01 (approximately 10^6^ cells/ml), respectively. A negative control without inoculum was incubated to account for any potential abiotic degradation of IAA. After 1 week of incubation, the cell-free supernatants were collected and stored at -20°C subsequent IAA concentration measurements. For IAA analysis, 100 μl of freshly prepared Salkowski’s reagent^113^ (12 mg/ml FeCl_3_ in 7.9 M H_2_SO_4_) was added to the same volume of 1/5X diluted samples in flat-bottom 96-wells microplates and incubated for 30 min at room temperature (23-26°C) in the dark. A_540_ absorbance was read using a microplate reader (iMarkTM, Bio-Rad). IAA was used as standard, although other indole acids including IPyA and indole-lactic acid could technically interfere. Calibration curves were included using 10-100 μM IAA in T-N medium. In these samples, ammonium content was measured using Nessler’s reagent,^37^ prepared mixing equal volumes of reagents A and B (MERCK 109011 and 109012, respectively). 100-μl samples of cell-free supernatants were transferred to flat- bottom 96-wells microtiter plates. A volume of 100 μl of freshly prepared Nessler’s reagent mixture was added, incubated for 2 min and A_410_ was read using a microplate reader (iMark, Bio-Rad). Ammonium calibration curves containing at least ten points of known concentrations of NH_4_Cl, ranging from 50 to 1,000 μM, were included in every measurement and samples were diluted as needed.

#### Cell quantification by quantitative PCR

The simultaneous quantification of *Chlamydomonas reinhardtii* and *Methylobacterium aquaticum* cell number was inferred by the quantification of the algal- and bacterial-specific single-copy genes as described in Calatrava et al.^37^ In brief, 213 bp of the *Chlamydomonas* centrin gene (Cre11.g468450.t1.2) were amplified using primers Cen1CreU and Cen1CreL and 239 bp of the *Methylobacterium rpoB* gene (Maq22A_c27070) were amplified using primers rpoBMaqU and rpoBMaqL. The sequences of these primers are indicated in the key resources table. For each qPCR run, 1 μl of each standard containing 1,010 copies/μl (centrin and *rpoB*) was serially 10-fold diluted, from 10^9^ to 10^1^ copies, and loaded in the same qPCR plate to quantify gene copies. qPCR was performed using SsoFast EvaGreen Supermix (Bio-Rad), and run and detected in MyiQTM2 (Bio-Rad) detection system. The conditions used for qPCR were: initial denaturation at 98°C for 2 min and 40 cycles of 5 s of denaturation at 98°C and 10 s of annealing and extension at 61°C.

The gDNA from the algal and bacterial samples was extracted as described in Calatrava et al.^37^ One ml of culture was harvested by centrifugation for 5 min at 3,000 x *g* and the pelleted cells were resuspended in 800 μl of lysis buffer (50 mM Tris·HCl pH 8.0; 0.3 M NaCl; 5 mM EDTA, pH 8.0; 2% sodium dodecyl sulfate). Then, samples were frozen and stored at -80°C until processed for phenol-chloroform extraction. Samples were thawed at 4°C and extracted using an equal volume of a phenol solution containing phenol:chloroform:isoamyl alcohol (25:24:1) saturated with 50 mM Tris·HCl, and vortexing vigorously for 1 min. Then, the samples were centrifuged for 5-10 min at 15,000 x *g* to separate both phases. The aqueous phase was transferred to a new tube and the extraction step with phenol solution was repeated for 2-3 times until no interphase was observed. Then, a last extraction step with chloroform was performed. DNA precipitation was achieved with 0.9 volumes of isopropanol and incubating for 1 hour at room temperature and then centrifuged for 30 min at 15,000 x *g*. The obtained gDNA was treated with RNase H (Promega). Nucleic acids concentration was quantified spectrophotometrically using NanoDrop™ (Thermo Scientific™).

### Quantification and statistical analysis

Data represent averages ± Standard Deviation. T-tests were performed using GraphPad Prism 6 with α<0.05 (∗), α<0.005 (∗∗); α<0.001 (∗∗∗), and three biological replicates (n=3) unless otherwise indicated in the figure caption. Data show a representative experiment and biological replicates were inoculated from independent pre-cultures and run in parallel.

## Supplemental information

Document S1. Figures S1–S9 and Tables S1–S3

Figure360. An author presentation of Figure 4
