# Supplementary material for: Genetic evidence for algal auxin production in Chlamydomonas and its role in algal-bacterial mutualism
Source: iScience. 2023 Dec 16;27(1):108762. doi: 10.1016/j.isci.2023.108762 (PMC10805672; doi:10.1016/j.isci.2023.108762)
Supplement: Document S1. Figures S1–S9 and Tables S1–S3 [file mmc1.pdf]

iScience, Volume *27*

Supplemental information

Genetic evidence for algal auxin production in *Chlamydomonas* and its role in algal-bacterial mutualism

Victoria Calatrava, Erik F.Y. Hom, Qijie Guan, Angel Llamas, Emilio Fernández, and Aurora Galván

Table S1. Expected α-keto acids produced by LAO1 activity for the 20 proteinogenic amino acids, related to *Introduction*.

L-Amino acid	α-Keto acid
L-Tryptophan	Indole-3-pyruvic acid*
L-Phenylalanine	Phenyl-3-pyruvic acid*
L-Methionine	2-Oxo-4-thiomethylbutanoic acid
L-Leucine	**α**-Ketoisocaproic acid
L-Isoleucine	3-Methyl-2-oxovaleric acid
L-Tyrosine	4-Hydroxyphenylpyruvic acid
L-Valine	**α**-Ketoisovaleric acid
L-Glutamine	2-Keto-glutarate
L-Serine	3-Hydroxypyruvic acid
L-Lysine	**α**-Keto-**ε**-aminocaproate
L-Asparagine	Oxaloacetamide
L-Alanine	Pyruvic acid
L-Arginine	2-Oxo-5-guanidinopentanoic acid
L-Histidine	Urocanate
L-Glutamate	Ketosuccinic acid
L-Aspartate	Oxaloacetate
L-Threonine	2-Hydroxy-2-oxobutanoate
Glycine	Glyoxylate
L-Proline	-
L-Cysteine	3-Mercaptopyruvic acid

*Auxin precursor

Table S2. Determination of putative generation of indole-3-acetic acid (IAA) from indole-3-pyruvic acid (IPyA) by algal cells and chemical conversion by hydrogen peroxide, related to Figure 1.

	IAA (mM)
IPyA (standard)	N.D.
IPyA + *Chlamydomonas*	N.D.
IPyA + H_2O_2 (without cells)	N.D.

IPyA (1 mM) was incubated with nitrogen-starved *Chlamydomonas* wild-type cells (5x10^6 cells/ml) or with hydrogen peroxide (1 mM) for 48 h. IAA was analyzed by HPLC in the cell-free supernatants. N.D., not detected (i.e., < 0.125 mM) The basal solution used was T-N media (see methods section)

Table S3. Ammonium was not detected in the supernatant of Methylobacterium spp. in IAA, related to Figure 3. Mono-cultures were incubated in N-free media supplemented with 500 μM of IAA for five days and ammonium was assayed in the cell-free supernatants using Nessler reagent. N.D., not detected. *Methylobacterium* spp. examined: *M. oryzae (Mory); M.* sp. 88A *(M88A); M. organophilum (Morg); M.* sp. M017 *(M017)*; *M. marchantiae (Mmar); M. hispanicum (Mhis); M. nodulans (Mnod); M. aquaticum (Maqu); M. aerolatum (Maer); Methylorubrum* (formerly *Methylobacterium) extorquens* (*Mext*).

Methylobacterium spp.	Ammonium
Mory	N.D.
M88A	N.D.
Morg	N.D.
M017	N.D.
Mmar	N.D.
Mhis	N.D.
Mnod	N.D.
Maqu	N.D.
Maer	N.D.
Mext	N.D.

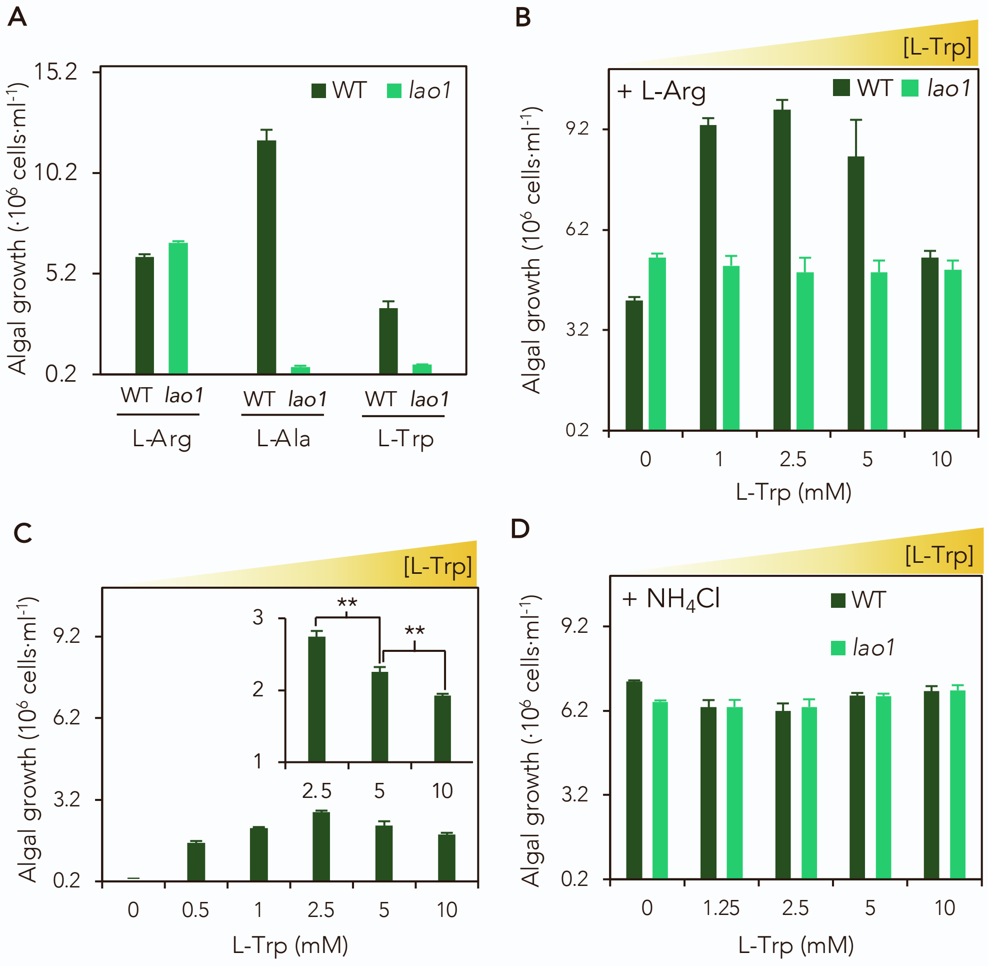

Figure S1. Impact of L-tryptophan and LAO1 in *Chlamydomonas* growth on different nitrogen sources. *A*, growth on L-arginine, L-alanine and L-tryptophan (8 mM) as the sole nitrogen source after three (L-Arg and L-Ala) and seven days (L-Trp). *B*, wild-type (WT) and *lao1* mutant (*lao1*) growth on 4 mM L-arginine supplemented with L-Trp for 3 days. *C*, WT growth on different concentrations of L-Trp as the sole nitrogen source for seven days. D, WT and *lao1* mutant cells grown on ammonium (4 mM) supplemented with L-Trp for 3 days. WT and *lao1* mutant were cultured at an initial concentration of $0.2x10^6$ cells·ml^{-1}. Data are means (SD) of three biological replicates. Asterisks indicate statistically significant differences compared to the control (T-test: n=3; α=0.05).

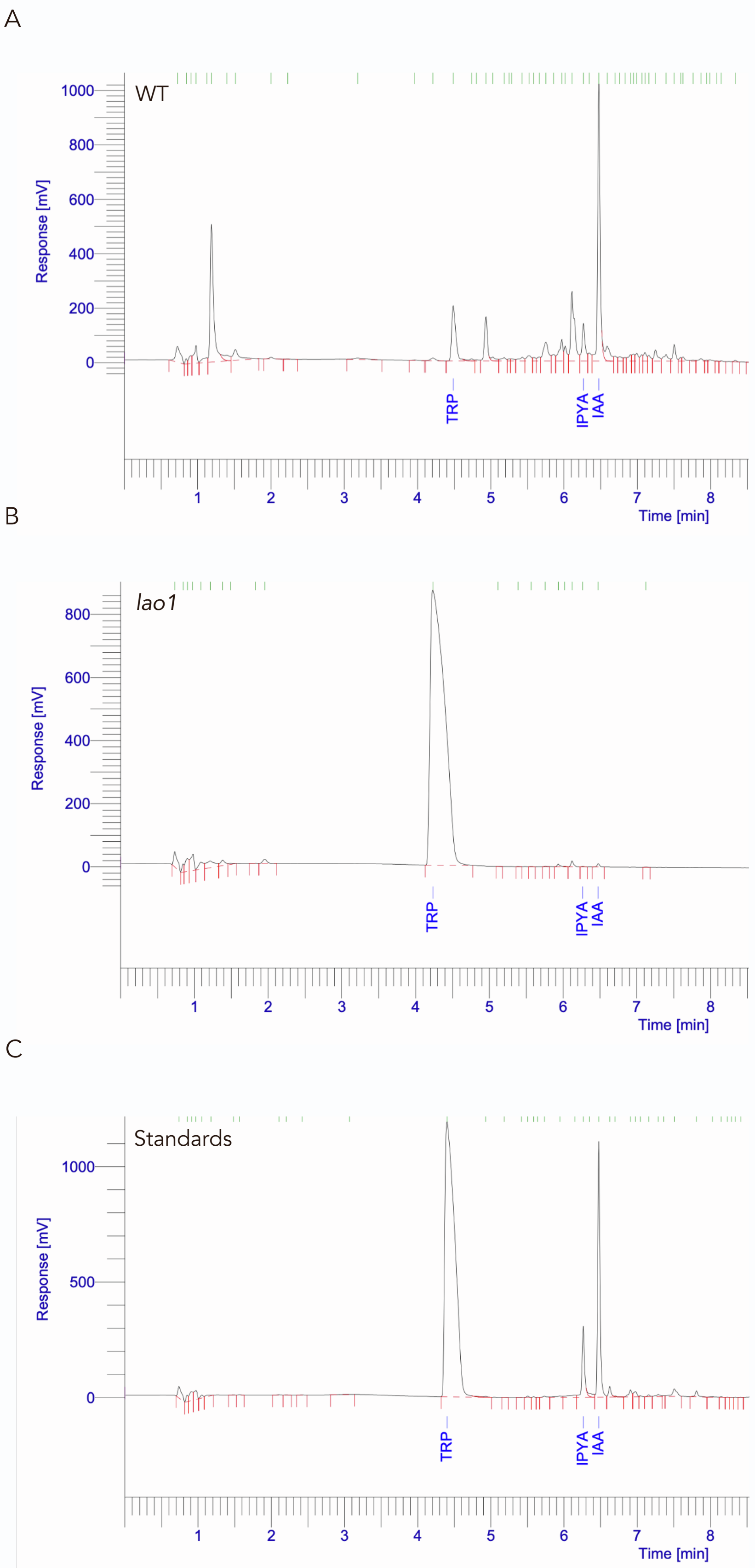

Figure S2. HPLC chromatograms resulting from the *Chlamydomonas* WT and *lao1* mutant supernatants corresponding to the results obtained in Figure 1. Wild-type (A) and *lao1* mutant (B) cells were incubated for 48 h in nitrogen-free medium supplemented with 5 mM L-tryptophan. Initial cell concentration was $5x10^6$ cells·ml^{-1}. A representative chromatogram for the three biological replicates is shown. C, standard mix of 5 mM L-Tryptophan (TRP), and 1 mM of indole-pyruvic acid (IPYA) and indole-3-acetic acid (IAA).

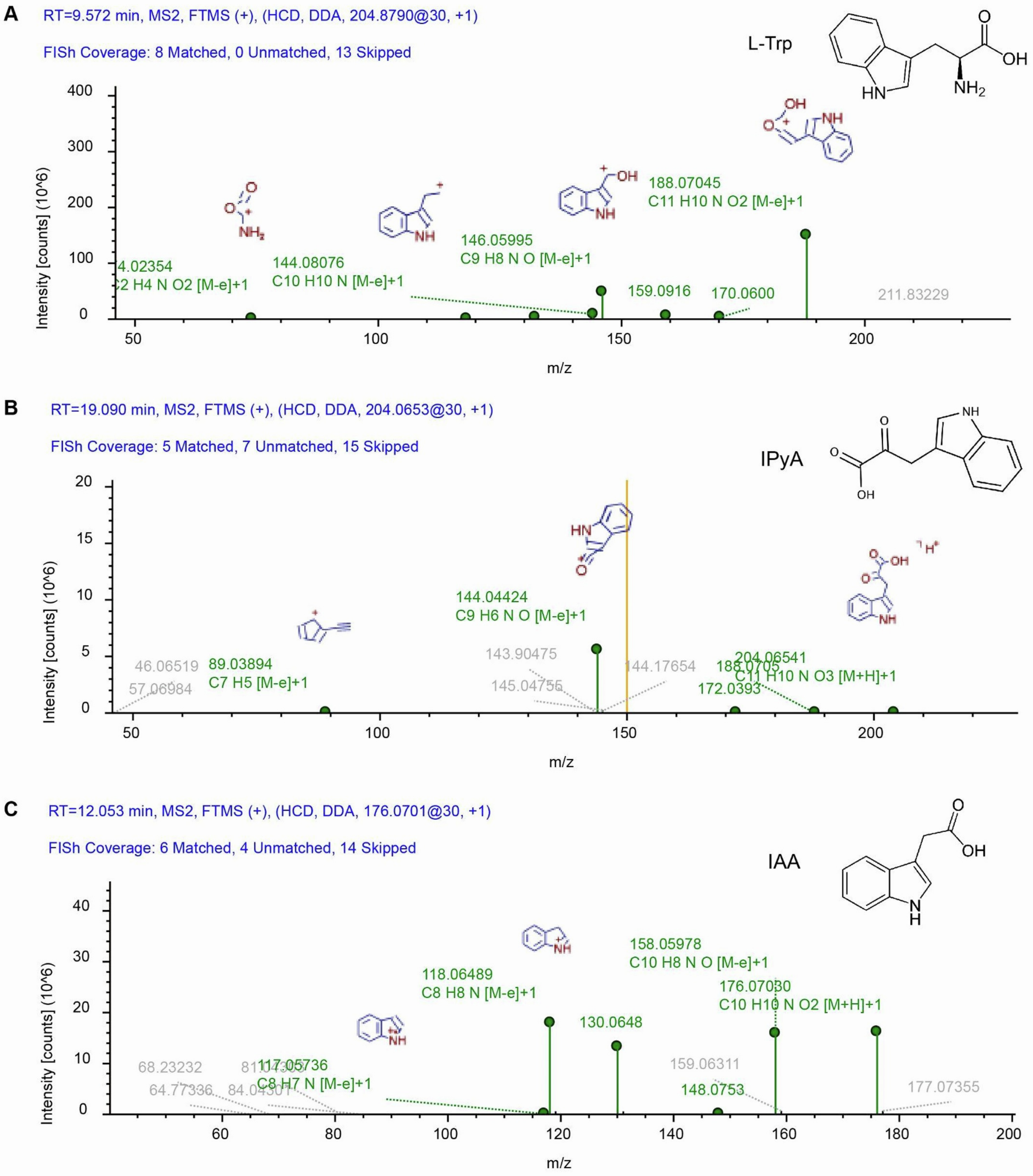

Figure S3. Identification of L-Trp, IPyA and IAA with HPLC-MSMS, related to Figure 1. The MS2 match was performed with Fragment Ion Search using Compound Discoveror 3.1 (Thermo Fisher Scientific, San Jose, USA), green dots represent MS2 matchings. (A) MS2 matches of L-Trp. (B) MS2 matches of IPyA. (C) MS2 matches of IAA.

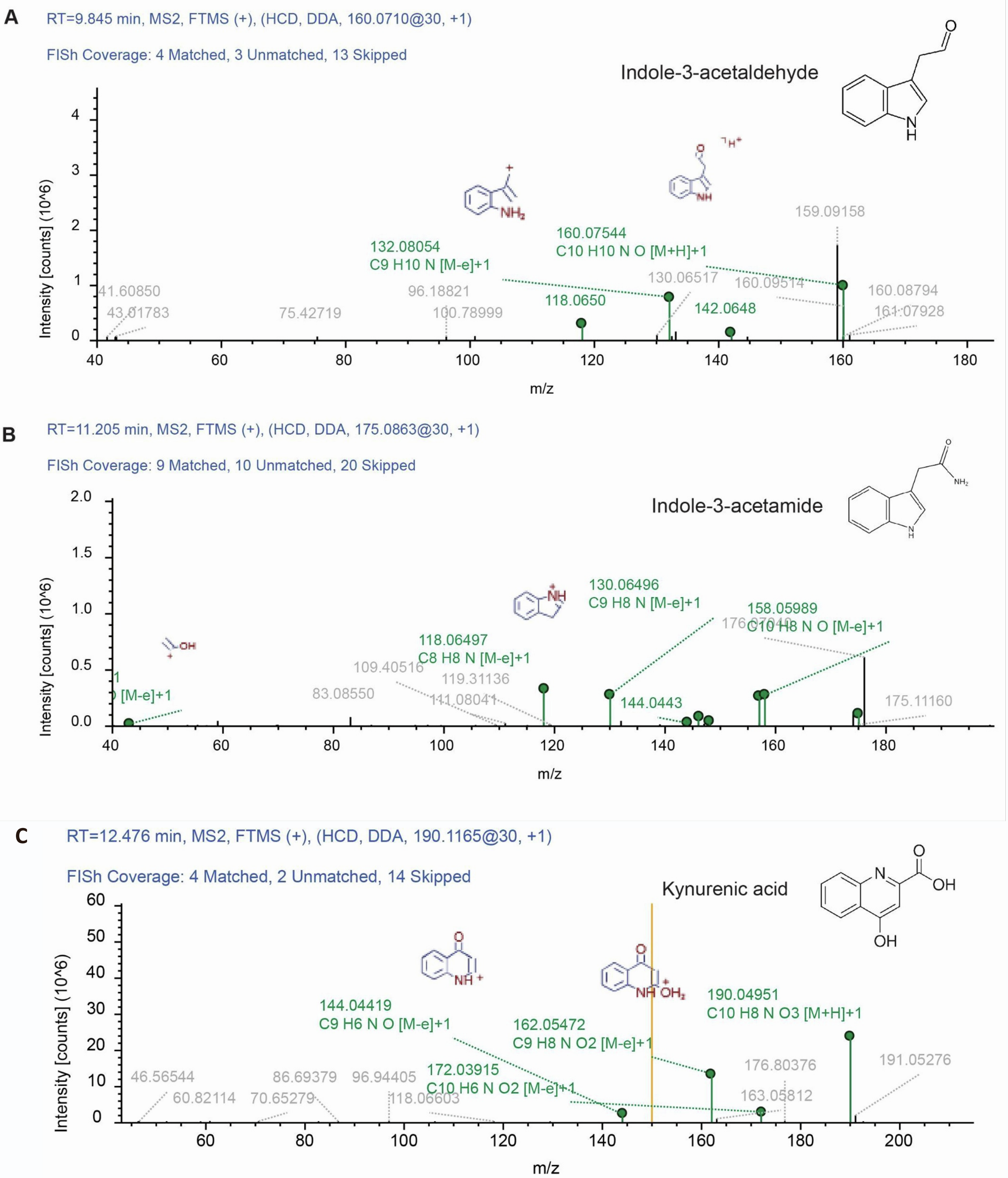

Figure S4. Identification of indole-3-acetaldehyde and indole-3-acetamide with HPLC-MSMS. The MS2 match was performed with Fragment Ion Search using Compound Discoveror 3.1 (Thermo Fisher Scientific, San Jose, USA), green dots represent MS2 matchings. (A) MS2 matches of indole-3-acetaldehyde. (B) MS2 matches of indole-3-acetamide. (C) MS2 matches for kynureic acid.

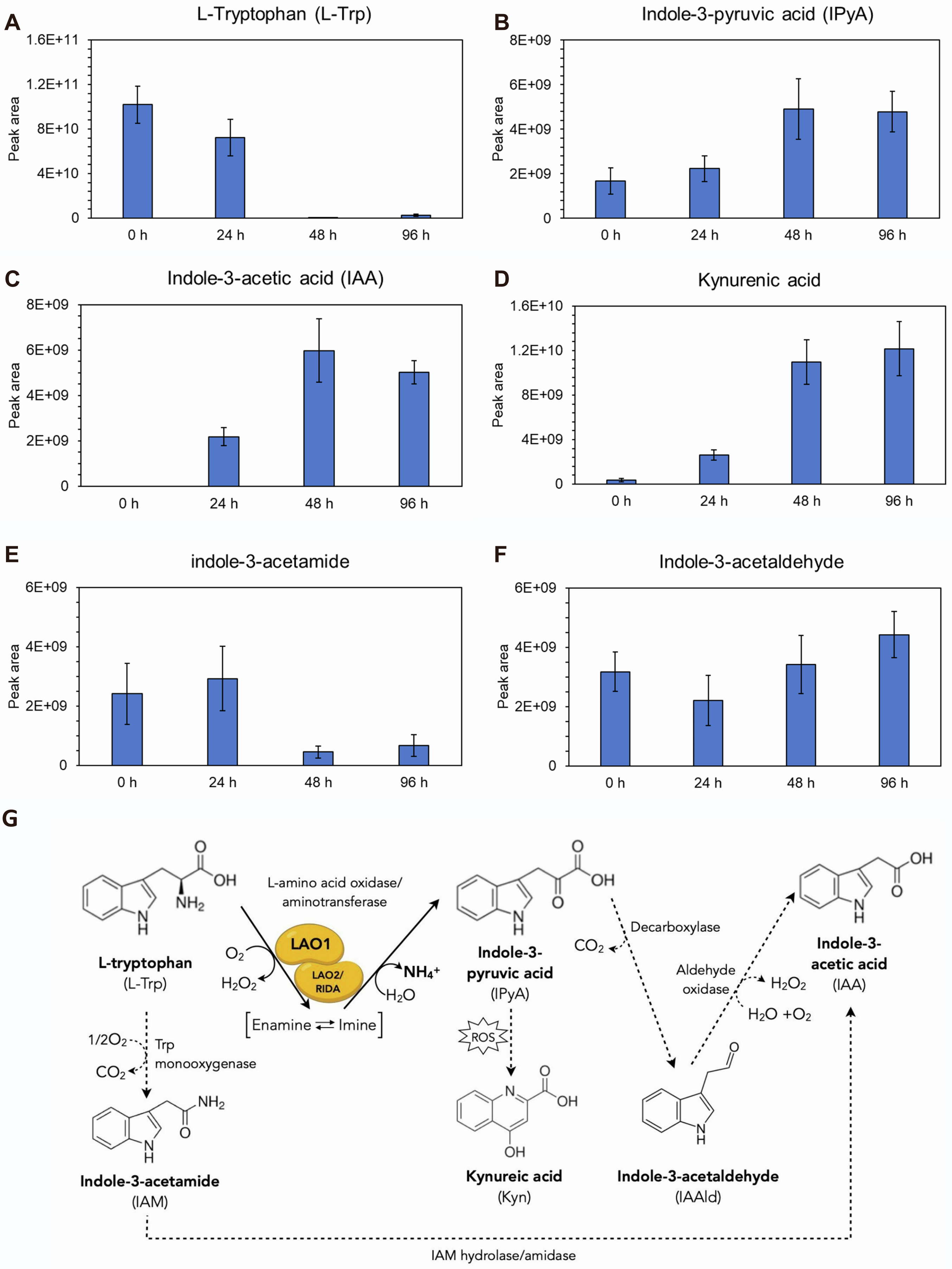

Figure S5. Peak areas of 6 identified compounds using HPLC-MSMS. The bars represent average peak areas in the group and the error bars represent standard deviations. (A) L-Trp changes during incubation. (B) IPyA changes during incubation. (C) IAA changes during incubation. (D) Kynurenic acid changes during incubation. (E) Indole-3-acetamide changes during incubation. (F) Indole-3-acetaldehyde changes during incubation. (G) L-Trp-dependent IAA biosynthesis pathway via IPyA intermediate and other intermediates that were detected in this work.

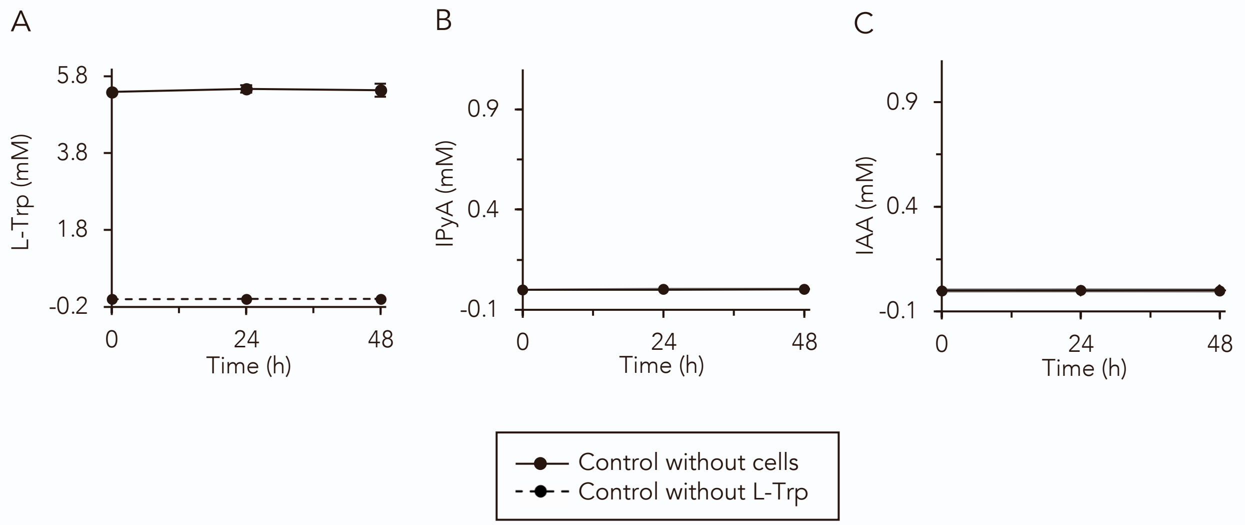

Figure S6. Controls for *Chlamydomonas* biosynthesis of indole-3-acetic acid (IAA) from L-tryptophan via indole-3-pyruvic acid (IPyA) pathway, related to Figure 1. A control without cells (with L-tryptophan) and other without L-tryptophan (with wild-type cells) were incubated for 48 h in nitrogen-free medium to discard the potential abiotic generation of indole-pyruvic acid (IPyA) or indole-3-acetic acid (IAA), or its biological but L-tryptophan-independent production. L-Tryptophan (A), IPyA (B) and IAA (C) were quantified in the cell-free supernatant/medium using HPLC. Data are means (SD) of three biological replicates.

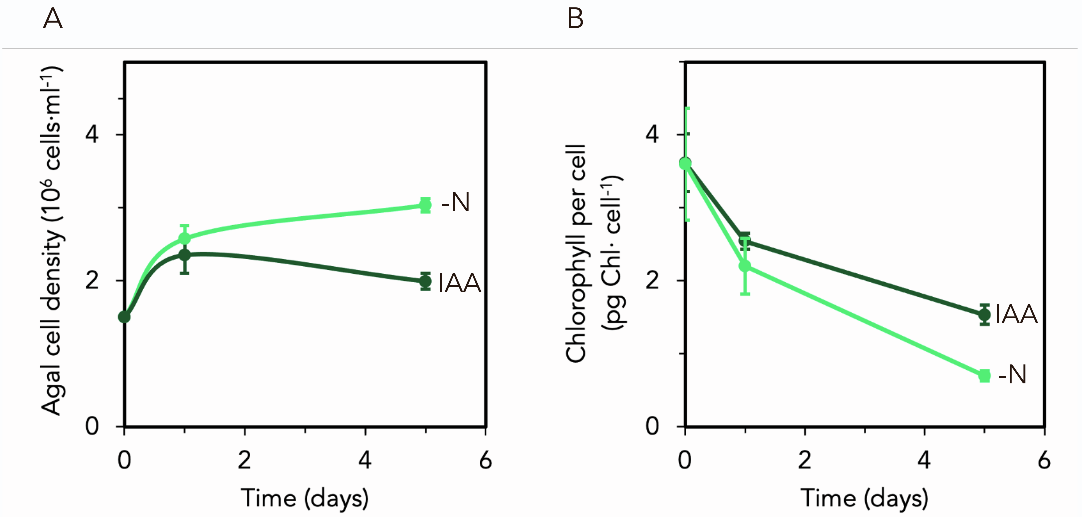

Fig. S7. Effect of IAA in *Chlamydomonas lao1* cell multiplication and chlorophyll content during nitrogen deprivation in the *lao1* mutant, related to Figure 2D. *Lao1* mutant cells were incubated in nitrogen-free media (–N) or supplemented with 500 μM indole-3-acetic acid (IAA). The algal cell density (A) and chlorophyll content (B) were determined at the at the indicated times

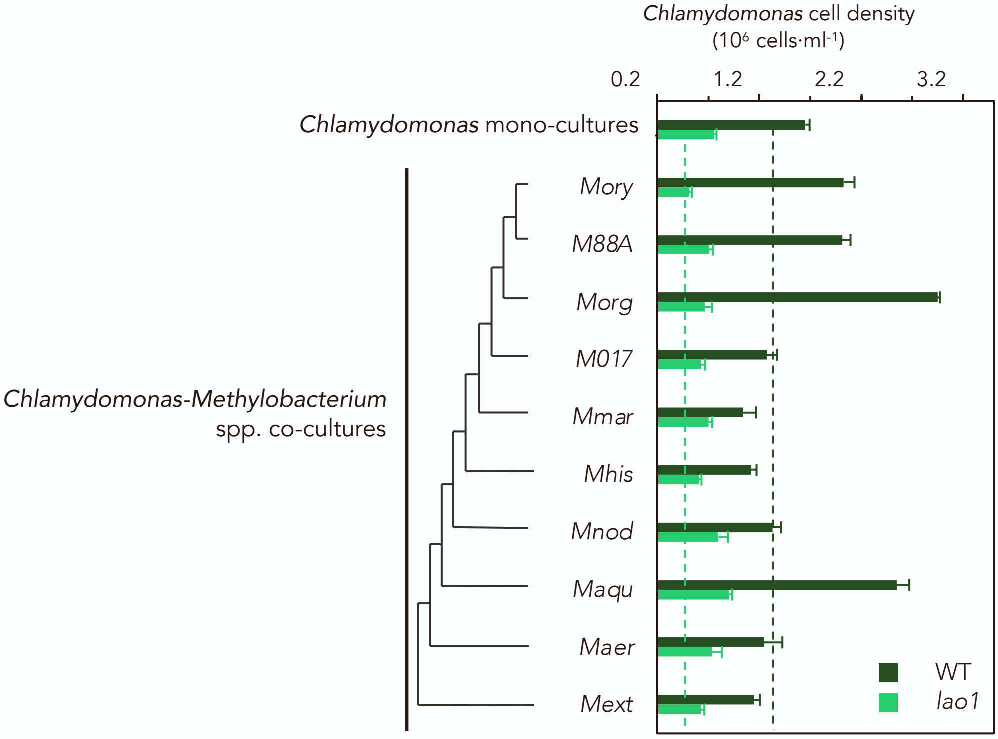

Figure S8. Algal growth during methylobacterial co-culture on L-tryptophan, related to Figure 3. *Chlamydomonas* cell concentration was determined after seven days of growth on 4 mM of L-tryptophan as the sole nitrogen source. Maximum likelihood tree was built using 16S rDNA sequences. *Mory, Methylobacterium oryzae; M88A, Methylobacterium* sp. 88A*; Morg, M. organophilum; M017, Methylobacterium* sp. M017*; Mmar, M. marchantiae; Mhis, M. hispanicum; Mnod, M. nodulans; Maqu, M. aquaticum; Maer, M. aerolatum; Mext, Methylovorus extorquens* (previously known as *Methylobacterium extorquens)*. WT, wild-type strain; *lao1*, *lao1* mutant strain. Data are means (SD) of three biological replicates.

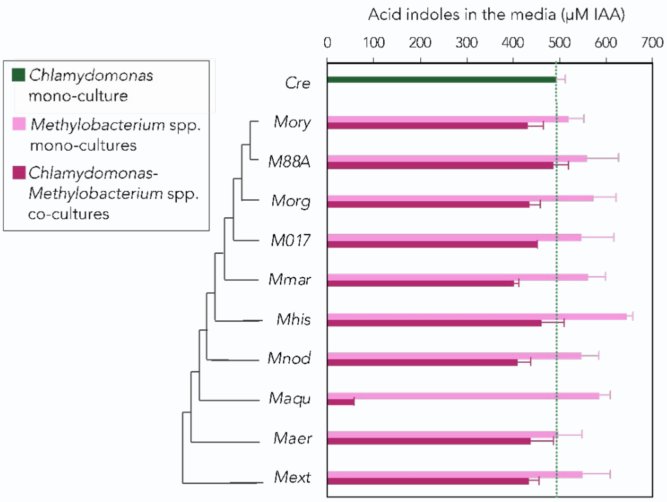

Dunn's multiple comparisons test	Rank sum diff.	Significant?	Sum mary	Adjusted P Value
Cre vs. *Methylobacterium* spp.	-9.000	No	ns	0.1017
Cre vs. *Cre-Methylobacterium* spp.	9.000	No	ns	0.1017
Methylobacterium spp. vs. *Cre-Methylobacterium* spp.	18.00	Yes	****	<0.0001

Figure S9. IAA degradation by Chlamydomonas and Methylobacterium spp. mono- and co-cultures, related to Figure 3. Algal and bacterial mono- and co-cultures were incubated on N-free media supplemented with 500 µM of IAA for five days. (*A*) Indole concentration in the cell-free media was determined using the Salkowski reagent (see *Materials and Methods*). *Methylobacterium* spp. examined: *M. oryzae (Mory); M.* sp. 88A *(M88A); M. organophilum (Morg); M.* sp. M017 *(M017)*; *M. marchantiae (Mmar); M. hispanicum (Mhis); M. nodulans (Mnod); M. aquaticum (Maqu); M. aerolatum (Maer); Methylorubrum* (formerly *Methylobacterium) extorquens* (*Mext*). Initial cell concentrations were 10^6 cells/ml for *Chlamydomonas* and A_{600} of 0.01 for *M. aquaticum* (approximately 10^6 cells/ml). (*B)* Dunn's multiple comparison test.
